# The Diagnosis of Cystic Fibrosis in Adult Age. Data from the Italian Registry

**DOI:** 10.3390/diagnostics11020321

**Published:** 2021-02-16

**Authors:** Rita Padoan, Serena Quattrucci, Annalisa Amato, Vincenzo Carnovale, Donatello Salvatore, Marco Salvatore, Giuseppe Campagna

**Affiliations:** 1 Regional Support Center for Cystic Fibrosis, Department of Pediatrics, Children’s Hospital–ASST Spedali Civili, University of Brescia, 25123 Brescia, Italy; 2 Italian Cystic Fibrosis Registry, 00162 Rome, Italy; Serena.Quattrucci@gmail.com (S.Q.); vincenzo.carnovale@unina.it (V.C.); saverdon@gmail.com (D.S.); marco.salvatore@iss.it (M.S.); gius.campagna@gmail.com (G.C.); 3 Italian League of Cystic Fibrosis–ONLUS, 00198 Rome, Italy; amatoann@libero.it; 4 Cystic Fibrosis Center, Adult Unit, Department of Translational Medical Science, University of Naples “Federico II”, 80138 Naples, Italy; 5 Cystic Fibrosis Center, Hospital San Carlo, 85100 Potenza, Italy; 6 National Center for Rare Diseases, Undiagnosed Rare Diseases Unit, Istituto Superiore di Sanità, 00162 Rome, Italy; 7 Department of Medical-Surgical Sciences and Translational Medicine, Faculty of Medicine and Psychology, “Sapienza” University, 00198 Rome, Italy

**Keywords:** cystic fibrosis, epidemiology, registry, diagnosis, adult diagnosis of cystic fibrosis, CFTR gene, sweat test, genotype, CFTR related disease

## Abstract

Cystic Fibrosis (CF) registries are an essential resource of epidemiological and clinical data. Although the median age at diagnosis is usually reported in the first months of life, a minority of individuals is diagnosed during adulthood. The aim of this study was to describe demographic, genetic, and clinical characteristics of this subgroup of the Italian CF population by using data from the Italian CF Registry (ICFR). Patients ≥18 years at diagnosis were selected and clinical data at diagnosis were analyzed from the 2012–2018 ICFR data (Cohort A). Subjects with diagnosis ≥18 years were selected from 2018 ICFR dataset (Cohort B) to describe their clinical status. In 2012–18 the incidence of late diagnosis was 18.2%, whereas, in 2018, the prevalence of patients diagnosed ≥18 years was 12.54%. The median age of late diagnosis was 36.2 years, ranging from 19.0 to 68.3. The male patients were diagnosed because of infertility in the 45.9% of cases. Median sweat chloride value (SCL) was 69 mmol/L (range 9–150). *F508del* mutation accounted for 28.3% of alleles. A wide variability in respiratory function was present with a median percent predicted Forced Expiratory Volume in the first second (ppFEV_1_) of 90.8% (range 20–147%). Low prevalence of pancreatic insufficiency (25%) and of *Pseudomonas aeruginosa* (*Pa*) infection (17%) suggest a mild CF phenotype in the majority of patients. The assessment of the clinical status in the 2018 dataset and the comparison between genders showed a greater nutritional and respiratory impairment in females. Further studies are needed to clarify the importance of a true diagnostic delay or of late onset of CF symptoms.

## 1. Introduction

Cystic Fibrosis (CF) is an autosomal genetic disease caused by alterations in the cystic fibrosis transmembrane conductance regulator (CFTR) gene leading to a dysfunctional protein and usually characterized by lung disease, digestive symptoms due to pancreatic insufficiency, male infertility due to absence of vas deferens, and increased chloride loss by sweat [1]. The basic therapies for CF patients, facilitating the clearance of the mucus from the lungs, the prevention and treatment of bronchial infection, the correction of pancreatic insufficiency and undernutrition, and saline supplementation, have resulted in improvements in their clinical outcomes, with a median life expectancy now older than 40 years [1].

CF registries are essential resources of epidemiological and clinical data, as well as are fundamental tool to plan CF public health services and research.

With the worldwide spread of CF newborn screening (NBS) [2], CF is usually diagnosed in the first months of life. Data from international CF registries report a median age at diagnosis at around 4 months old (Italy: 4.2mo; Europe: 4mo; USA: 3mo), whereas the range of age at diagnosis is very wide, reaching even the eighth decade of life [3,4,5].

NBS health policies offer the possibility to the majority of CF patients to be identified and followed within dedicated and specialized CF Centers during the first months of life. Despite this, about 80–90% are diagnosed within 10–12 years of life, and a minority of individuals are identified only in adulthood, i.e., late CF diagnosis [3,4,5].

Several reports include description of the diagnoses of CF during adulthood in patients suffering from CF-like symptoms, such as bronchiectasis or azoospermia [6,7,8,9,10,11].

To date, although relevant interest is dedicated by the scientific community to adult patients with CF-like symptoms [12], very limited information is available on this population within national CF registries [3,4,5].

The aim of this study was to characterize Italian patients who received a CF diagnosis ≥ 18 years (late diagnosis), using data from the Italian CF Registry (ICFR).

### Italian Cystic Fibrosis Registry

CF patients in Italy are taken in charge by dedicated Italian CF regional referral and support Centers (established by Italian law 548/93); these Centers share information on patients with the ICFR (www.registroitalianofibrosicistica.it/ (accessed on 10 January 2020)) coordinated by the Italian Institute of Health and all Italian stockholders involved in CF clinics, diagnosis, and research. A total of 29 CF Centers annually sent their data to ICFR, and, in 2018, 5.501 CF patients were registered in the ICFR. The aims of the ICFR are (i) to analyze the medium and long term clinical and epidemiological trends of the disease; (ii) to identify the main health care needs at regional and national level to contribute to the Health Care programmes and to the distribution of resources; and (iii) to compare Italian data with international ones.

Median age of patients included in ICFR increased in a 10 years period long (2010–18; 17 years in 2010 versus 21.2 years in 2018). Range of patients’ age is from first month of life to 81.5 years. Data from 2018, shows that pediatric death is a very rare event and that a very low percentage of pediatric population is characterized by severe lung disease (FEV1% < 40). Prevalence of adult patients increased (56.4%). On the opposite end, age at diagnosis decreased (3.8 months in 2018) and median age at death (transplanted patients not included) was 35.8 years in 2018.

## 2. Methods

Data were collected from the ICFR, which includes information from about 95% CF Italian population. A retrospective observational study was performed by selecting two distinct cohorts with a late CF diagnosis:

Cohort A (*n* = 204): including *demographic*, *genetic*, and *clinical features at diagnosis* data from patients with a late diagnosis in the period 2012–2018 and describing each patient clinical status at the moment of CF diagnosis.

Cohort B (*n* = 690): including *demographic*, *genetic*, and *clinical features* in 2018 CF registry data to describe the actual clinical status in patients diagnosed as CF in adulthood age.

In regard to chloride sweat value (SCL), 29 mmol/L was considered as non-CF limit value, while “borderline” values ranged from 30 mmol/L to 59 mmol/L, as stated by international guidelines [13].

CFTR2 definitions [14] were adopted for genotype analyses and alleles categorization into *CF-causing* variant, variant with *variable clinical consequences* (VCC), and *no-CF variant* [15]. The “*unknown*” label was adopted in case of variants not included in the CFTR mutation database (www.genet.sickkids.on.ca (accessed on 10 January 2020)) or in the CFTR2 mutation database (https://cftr2.org (accessed on 10 January 2020)), in case of CFTR2 definition of “*unknown clinical consequences*”, or when an allele was reported as not identified.

A comparison between genders was carried out on patients of cohort B, with respect to respiratory function, using percent predicted Forced Expiratory Volume in the first second (ppFEV_1_) and percent predicted Forced Vital Capacity (ppFVC), and nutritional status, using Body Mass Index (BMI).

All patients, or their legal representatives, gave informed consent for inclusion in the ICFR. The present data analysis project was reviewed by the Scientific and Steering Committees of the ICFR, and anonymous patient data were provided for analysis (date of approval: 2020, 10 February). The terms of use of the provided data were governed by the Italian law in accordance with European data protection legislation.

### Statistical Analyses

Continuous variables were showed as median (minimum to maximum) or 95% Confidence Interval (CI). Categorical variables were expressed as absolute frequencies and percentages *n* (%). The normality of the continuous variables was tested by Shapiro–Wilk test. The Mann–Whitney test was used to compare sex against the continuous variables shown in Table 3. The association between sex and categorical variables was evaluated by chi square test or Fisher exact test when appropriate. The z test was applied for to compare the sex to BMI classes. A probability level of *p* < 0.05 was considered statistically detectable. Statistical analyses were conducted using SAS v.9.4. (SAS Institute Inc., Cary, NC, USA).

## 3. Results

### 3.1. Cohort A

#### 3.1.1. Patients Diagnosed in Adulthood from 2012 to 2018

In the study period, the incidence of new late diagnoses was 18.2% (204/1122), and prevalence of male sex was 54.5% (*n* = 111).

The median age at diagnosis was 36.21 years, ranging from (19.00 to 68.33).

In the male subgroup (111 subjects) the presence of infertility led to a CF diagnosis in 51 (45.9%). In regard to the remaining subjects (60 males, 93 females), CF was diagnosed due to the presence of CF-like symptoms (in 70% of 153 subjects), and CF familiarity (30% of 153 subjects).

Clinical features of adult new CF diagnoses in cohort A are presented in Table 1.

Median chloride sweat value (SCL) value was 69 mmol/L (range: 9–150 mmol/L). SCL was in the normal (non-Cystic Fibrosis (CF)) range in 10 subjects (5.5%), while, in 45 subjects (25%), it was in the borderline range (30–59 mmol/L).

The genetic analysis was reported for 198 out of 204 identified patients. *F508del* mutation accounted for 28.28% of alleles. Three patients were *F508del* homozygotes (1.5%), 106 were *F508del* heterozygotes (53.5%), and, in 89 patients, no *F508del* mutation was identified (44.9%).

Two CF-causing mutations were found in 70 subjects (35.3%), whereas 72 subjects (36.3%) were compound heterozygotes with one CF-causing allele and one varying clinical consequences (VCC) variant, and 8 (4%) with two VCC variants. Among the remaining 48 subjects: in 18 cases, genotypes were one CF-causing and one identified allele with unknown clinical consequences, and, in 23 cases, at least one allele was unidentified, while 7 cases bear at least one defined no-CF allele.

The most frequent genotypes were *F508del/5T*;*TG12* (22 cases) and *F508del/D1152H* (13 cases).

Table 2 reports the frequencies of the different alleles, and Table 2 shows the actual genotype frequencies divided by CFTR2 categories.

Clinical status of the patients in cohort A in the year of diagnosis was good: only 25 subjects had pancreatic insufficiency (PI) (12.2%). Malnutrition was rare (9% among females and 4% among males), while more than half of the subjects presented a good nutritional status (51.77% of females and 65.31% of males).

A wide variability in respiratory function was present. The median of predicted percent FEV_1_ (pp FEV_1_) was 90.77% (range: 19.78–147.03%), with the range from a severe (ppFEV_1_ < 20%) to a normal situation (ppFEV1 > 90%). Only 31 subjects (17.0%) had *Pseudomonas aeruginosa* (*Pa*) infection.

#### 3.1.2. CF Patients Diagnosed Due to Infertility in the 2012–2018 Period

Fifty-one males (45.9%) were diagnosed as CF patients in the 2012–2018 long period due to infertility. Median age at diagnosis was 37.35 years, with a range from 19.93 to 56.08 years. In the year of diagnosis, this cohort of subjects presented a good nutritional status (median BMI = 24.38), and none was malnourished; good pulmonary function (median ppFEV_1_ = 96.51%) was also observed, and a ppFEV_1_ less than 70% was reported in only 12% of subjects. Few patients had pancreatic insufficiency (7.84%) and chronic *Pa* infection (5.88%).

Genetic characterizaction of this group showed the presence of 2 CF-causing variants in 13 subjects without the F508del homozygous variant. In 21 patients, the combination of a CF-causing mutation with a VCC variant was identified, and, in 3 subjects, two VCC variants were identified. In 11 additional patients, a CF-causing mutation/variant with unknown clinical significance genotype was identified; another subject had two unknown variants, and two had at least one no-CF variant.

The most frequent genotype was *F508del/5T;TG12*, identified in the 23.5% of patients.

*F508del* accounted for 30.4% of alleles, while 17 other CF-causing variants accounted for the 27.4% (28/102 alleles), and six different VCC variants for the 26.5% (27 alleles); finally, for the 12.7% (13 alleles), it was reported that variants had not known clinical consequences (alterations not present in CFTR mutation database and/or in CFTR2 database or not identified).

#### 3.1.3. Mortality/Transplant

No deaths were recorded in this patient cohort, and only one female patient underwent a double lung transplant four years after diagnosis.

### 3.2. Cohort B

#### 3.2.1. Clinical Status in 2018 of Patients with a Late CF Diagnosis

A total of 690 patients (379 males, 54.9%) receiving a CF diagnosis in adulthood (CF late diagnosis) are included into the 2018 ICFR database, representing the 12.54% of the 2018 Italian CF population (5501 patients).

The current age of this cohort ranges from 20 to 86 years, with a median age similar in males and females (45.63 years and 45.87 years, respectively). In regard to age at diagnosis, its range varied from 18 years to the eighth decade of life (18.02–77.62 years), and females were diagnosed at a median age similar to that of males (31.56 versus 33.69 years) (*p* = 0.19). A minority of patients (*n* = 28; 4.06%) were diagnosed over the age of 60 years, due to respiratory symptoms in all but four, who were diagnosed for positive family history.

Male infertility was the main cause of CF diagnosis in males (161 subjects, 42,48%). Diagnosis was made in the remaining subjects accordingly to CF-symptoms, mainly respiratory, in 62.52% of cases, and/or to familiarity for the disease in 20%.

Median SCL at diagnosis was 77.20 mmol/L (range: 9–150 mmol/L).

Among patients included in cohort B, only 2.9% (*n* = 20) of subjects were homozygous for *F508del* mutation; 55.51% (*n* = 383) was *F508del* heterozygous; in 41.45% (*n* = 286) of subjects, the *F508del* allele was not identified.

In Table 3, the clinical features in 2018, are shown for males and females in this cohort of patients.

In regard to nutritional status, a statistically significant difference (*p* < 0.0001) between males and females was observed, with the median BMI higher in males (24.22 kg/m^2^) than in females (22.22 kg/m^2^). Malnutrition (BMI < 18.5 kg/m^2^) was instead observed in 6 males (1.68%) and in 22 females (7.56%) (*p* < 0.0003). Moreover, 63.31% of males were overweight (BMI ≥ 23).

Median ppFEV_1_ value in female was significantly lower than that observed in males (73.93% versus 87.26%, respectively; *p* < 0.0001). A similar data was also evident for ppFVC values (Table 3). In particular, a significantly lower percentage of women had a ppFEV_1_ in the normal range as compared to men (25.53 % versus 44.81%, respectively; *p* < 0.0001), and a significantly higher percentage of females had a ppFEV_1_ < 40% (12.41%) than males (7.72%) (*p* = 0.049).

In parallel with the lung function analyses, we estimated the prevalence of the most important chronic infection within cohort B; *Pa* infection was significantly higher in females than in males (43.0% versus 28.4%, respectively; *p* < 0.0001), whereas the prevalence of *Staphylococcus aureus* colonization was similar in the two subgroups (Table 3).

PI was reported in a minority of patients (32.89%) without differences between genders.

#### 3.2.2. CF Subjects Diagnosed Due to Infertility, Patients in the 2018 Database

Males CF patients, diagnosed due to the presence of infertility, may have a milder phenotype at diagnosis (results from Cohort A); thus, we analyzed a subgroup (*n* = 161) of individuals from 2018 ICFR database diagnosed because of infertility (Table 4).

In this cohort of patients, excellent nutritional status (median BMI of 25) and good respiratory function (median ppFEV_1_ equal to 95.96% and ppFVC equal to 97.67%) were confirmed in 2018 at a median age of 44.46 years.

However, although median ppFEV_1_ and ppFVC observed in this group was > 95%, a minority of patients had a respiratory function lower than 70% (11.49%) or *Pa* chronic infection (12.58%).

#### 3.2.3. Comparison Between Gender: Female Versus Male Patients Not Diagnosed Because of Infertility

To better understand the suggested differences between genders shown in Table 3, a clinical characteristics comparison between the entire female group (*n* = 311) and the males (*n* = 218) after exclusion of those diagnosed due to infertility was performed (Table 5).

The statistically significant difference in median BMI values was confirmed (23.51 versus 22.22, *p* < 0.0001). In the male group, after exclusion of subgroup diagnosed because of infertility, the percentage of malnourished subjects increased (from 1.68% to 2.43%), with a concomitant reduction in the percentage of overweight males (from 63.31 to 55.83%) (Table 3 and Table 5).

The ppFEV_1_ and ppFVC differences observed were not maintained in the new analysis: median ppFEV_1_ values were 76.83 and 73.93 (*p* = 0.31), while median ppFVC were 87.82 and 85.24 (*p* = 0.20), respectively, for males and females. Pancreatic insufficiency frequency was higher in males than in females (44.5% versus 29.84%; *p* = 0.0006). No difference in percentage of *Pa* infection (39.81% versus 43%; *p* = 0.47) was observed.

#### 3.2.4. Genotype Characterization of Patients with Normal or Borderline Chloride Sweat Test

One hundred and thirty out of 690 subjects of cohort B had an SCL lower than 60 mmol/L (18.84%).

Seventeen out of 690 patients (2.46%) of cohort B had a normal SCL value (<30 mmol/L). Only 2 patients were characterized by the presence of CF-causing variants (genotypes *N1303K/3849* + *10kbC* > *T* and *F508del/R117C*); 9 patients had genotypes consisting of a CF-causing and a VCC variant. Three subjects are heterozygotes for a CF-causing variant and an unknown variant, and 2 patients had no-CF-causing variants and one an unidentified genotype.

Among the 34 alleles, six different CF-causing variants were identified in 47%, and three different VCC variants in 20%. For four CFTR alterations (12% of the alleles), there is no information in CFTR2 database, while a further three identified variants are labeled as non-CF alterations.

One hundred and thirteen patients out of 690 (16.3%) had an SCL value in the borderline range (30–59 mmol/L). In this group, 15 subjects had two CF-causing variants; 63 subjects were characterized by a genotype with a CF-causing mutation and a VCC variant, the most frequent genotype being *F508del/D1152H*, identified in 22 patients; 24 patients had at least one variant with unknown clinical significance; 5 patients were characterized by at least one no-CF variant.

In this subgroup, 22 different CF-causing variants accounted for 118 alleles (52.2%), and 14 different VCC variants accounted for 74 alleles (32.7%), while 28 alleles (12.4%) had no known clinical consequences (variants not present in CFTR mutation database and/or in CFTR2 database or with unknown clinical consequences or unidentified), and 6 (2.7%) alleles were classified by CFTR2 database as No-CF variants.

Table 6 shows the actual genotype frequencies stratified by CFTR2 categories in the subgroup with borderline SCL.

## 4. Discussion

The present study explored the demographic, genetic, and clinical characteristics of CF patients diagnosed in adulthood through the use of the data collected in the ICFR from 2012 to 2018. The data on nutritional status, respiratory function, and the prevalence of pancreatic insufficiency might suggest a milder CF phenotype in this specific subgroup.

Although CF diagnosis in adult patients and their needs arouse a great interest within the CF scientific community [12,16,17], limited information on this topic is still available in literature.

The ICFR data allowed us to describe characteristics of (i) a large cohort of subjects (*n* = 204) in the year they received their diagnosis (from 2012 to 2018), and (ii) an even larger cohort (*n* = 609), including clinical description of all the patients diagnosed in adulthood and included in the 2018 ICFR data.

CF patients diagnosed in adulthood represent 12.4% of all patients included in the ICFR; this data is in line with those previously reported from other registries (10–15%) [18,19].

Our results indicate that, in Italy, every year, a large proportion (about 20%) of new CF cases are diagnoses in adult life, up to the eighth decade of life.

About the 25% of these patients are aged less than 30 years at the time of diagnosis; these patients should have undergone NBS, in which a pilot program in Italy began at the end of the 70s and spread throughout the national territory in the following decades [20]. Unfortunately, CF NBS coverage in 1998 reached only 35.6% of the Italian neonatal population [21]. So far, we hypothesize that diagnostic delay could be due to the absence of activation of dedicated CF NBS programs within a specific region, rather than a negative screening or a positive neonatal screening followed by a negative or borderline sweat test. Last but not least, a small number of subjects is diagnosed later than 60 years of age, thus highlighting that a CF diagnosis should be considered during all human lifespan.

Familiarity is among the most important reasons of diagnosis in adulthood (30% of total). The data collected by the IRCF does not allow to investigate further the degree of kinship leading to the diagnosis of CF, although it can be assumed that there may be cascade diagnoses from an index case diagnosed at an earlier age [22].

Among our CF late diagnosed population, there are subjects with sweat chloride values lower than 60 mmol/L.

The evaluation of genotypes in subjects with a normal or borderline sweat test demonstrated the wide genetic variability, typical of the Italian population [23]. Only 15 out of 113 (13.27%) of subjects with a borderline SCL had two CF-causing mutations (Table 6). 37 out of 130 patients (28.46%), whose SCL was in normal or borderline range (<60 mmol/L), were not characterized from a genetic point of view. However, it is possible that the complete analysis of the CFTR gene by sequencing was not performed in all cases with non-pathological sweat test.

Numerous CFTR gene variants, characterized by variable or even unknown clinical consequences, were identified in patients with negative (<30 mmol/L) or borderline (30–59 mmol/L) sweat tests: this could explain the difficulty in diagnosing CF in adulthood [24] and the need for more widespread sensitive tests to define CF diagnosis, in case of a lacking confirmatory CFTR gene sequencing.

The in vivo activity of the mutated CFTR, such as the measurement of the nasal potential difference (NPD) or of the intestinal current (ICM) [25] or the in vitro study of the function of CFTR [26], could be useful in confirming CF diagnosis.

This study was not performed with the aim to evaluate the appropriateness of the CF diagnostic label in late diagnosis patients, but, as already stated in other studies [27], our results confirm the presence of patients who do not completely fulfill the criteria for being labeled as CF (see above), within Italian CF Registry. These data need to be further investigated.

The clinical conditions at the time of diagnosis are good for the majority of patients, both in terms of nutritional status and respiratory function, with median values of BMI and ppFEV_1_ within the normal range. A small percentage of patients had pancreatic insufficiency (13.16%), a respiratory function lower than 70 ppFEV_1_, and/or chronic *Pa* infection (<20%), or are malnourished (<10%). Moreover, confirming the overall satisfactory clinical status, only one out of the 204 subjects diagnosed in the period 2012–2018 underwent a double lung transplantation, and none died.

The subjects diagnosed for infertility presented excellent clinical conditions at diagnosis, and a significant quote is overweight, this representing an emerging clinical problem to be faced in CF patient care [28]. Among them, only 13 out of 51 subjects (25.49%) had genotypes with two CF-causing mutations. It is, therefore, possible that a fair number of subjects are actually CFTR-related disorder (CFTR-RD) (as male infertility in CF is because of Congenital Bilateral Absence of Vas Deferens (CBAVD)) incorrectly labeled as CF, as really the most frequently identified genotype in this group is *F508del/5T*; *TG12*.

The 2018 assessment of the clinical status in the cohort of subjects diagnosed in adulthood and the comparison between genders showed a greater nutritional and respiratory impairment in females, together with a higher frequency of chronic *Pa* infection. The results obtained in this large cohort confirm what has already been established [29] on the presence of a gender gap to the detriment of females.

Subsequently re-evaluating the difference between genders after excluding the males diagnosed by infertility, the observed differences in respiratory function (worse in females), and in the percentage of chronic *Pa* infection (higher in women), were not confirmed. On the contrary, the differences between sexes in nutritional status are maintained (worse in women).

Information on clinical and genetic data of subjects diagnosed for infertility suggests that the “infertile males” group could include both CVABD subjects and subjects suffering from classic CF forms. The latter patients were diagnosed because of infertility and not for the typical respiratory and pancreatic symptoms of the disease, thus indicating a real diagnostic delay in some patients.

## 5. Conclusions

The diagnosis of CF in adulthood is evident in our country, as well. Patients diagnosed in adulthood have peculiar genetic and clinical characteristics with a generally “mild” phenotype and often borderline or even negative sweat tests, posing serious difficulties in the diagnostic definition of CF or CFTR-RD (CBAVD). Many of them have been defined as CF based on clinical criteria suggestive for the disease, despite not having two CF-causing mutations and/or pathological sweat tests. It is, therefore, necessary that, when faced with the diagnostic suspicion of CF in an adult, whose classical laboratory data do not fully meet the CF diagnosis criteria, clinicians equip themselves with tools suitable for improving the diagnostic confirmation capacity, such as sequencing of the whole CFTR gene and in vivo or in vitro measurement of mutated CFTR activity.

Further studies are needed to clarify the weight of a true diagnostic delay or a late manifestation of CF symptoms in this patient cohort.

## Figures and Tables

**Table 1:** Characteristics of patients with late diagnosis in 2012–2018.

Characteristic	2012 to 2018 (*n* = 204) Median (Minimum to Maximum)
Sex (Female/Male) *	93 (45.59)/111 (54.41)
Age at diagnosis (years)	36.21 (19.00 to 68.33)
BMI (kg/m^2^)	23.44 (15.06 to 41.29)
BMI class for female *	
*<18.5*	*8 (9.41)*
*[18.5 to 22)*	*33 (38.82)*
*≥22*	*44 (51.76)*
BMI class for male *	
*<18.5*	*4 (4.08)*
*[18.5 to 23)*	*30 (30.61)*
*≥23*	*64 (65.31)*
FEV_1_ predicted (%)	90.77 (19.78 to 147.03)
FEV_1_ predicted class *	
*<40%*	*10 (5.68)*
*[40% to 70%)*	*28 (15.91)*
*[70% to 90%)*	*47 (26.70)*
*≥90%*	*91 (51.70)*
FVC predicted (%)	93.43 (25.21 to 139.34)
Sweat chloride (mmol/L)	69.00 (9.00 to 150.00)
Sweat chloride class *	
*<30*	*10 (5.46)*
*[30 to 59)*	*45 (24.59)*
*≥59%*	*128 (69.95)*
Pancreatic insufficiency *	25 (12.2)
Pseudomonas aeruginosa *	31 (17.03)
Staphylococcus aureus *	57 (31.15)

* data presented as *n* (%). In italic the data regarding the BMI and ppFEV_1_ classes.

**Table 2:** a. Frequency of cystic fibrosis transmembrane conductance regulator (CFTR) alleles in 198 subjects. b. Genotype frequencies per CFTR categories.

a. CFTR Variants	*n* (%)
F508del	112 (28.28)
5T; TG12	33 (8.33)
D1152H	26 (6.57)
G542X	13 (3.28)
N1303K	12 (3.03)
2789 + 5G > A	12 (3.03)
3849 + 10kbC > A	11 (2.78)
G85E	4 (1.01)
1717-1G > A	3 (0.76)
W1282X	3 (0.76)
R117H	2 (0.51)
Other identified	134 (33.84)
Other no-CF	8 (2.02)
Unidentified	23 (5.81)
TOTAL	396 (100.00)
**b. Genotype**	***n* (%)**
CF-causing/CF-causing	70 (35.35)
CF-causing/Variable Clinical Consequences	72 (36.36)
CF-causing/Unknown	35 (17.68)
CF-causing/No-CF	4 (2.02)
Variable Clinical Consequences/Variable Clinical Consequences	8 (4.04)
Variable Clinical Consequences/Unknown	1 (0.51)
Variable Clinical Consequences/No-CF	2 (1.01)
No-CF/No-CF	2 (1.01)
Unknown/Unknown	4 (2.02)
Total	198 (100.00)

**Table 3:** Cohort B: difference between variables in patients with a CF late diagnosis according to sex in 2018.

Characteristic	Male (*n* = 379) Median (95%CI)	Female (*n* = 311) Median (95%CI)	*p*
Age (years)	45.63 (44.87 to 47.46)	45.87 (44.38 to 48.13)	0.66
Age at diagnosis (years)	33.69 (32.55 to 34.58)	31.56 (30.48 to 32.97)	0.19
BMI (kg/m^2^)	24.22 (23.70 to 24.66)	22.22 (21.80 to 22.56)	**<0.0001**
BMI class for female and male *			
*<18.5*	*6 (1.68)*	*22 (7.56)*	**0.0003**
*BMI class for female* *[18.5 to 22)*	--	*114 (39.18)*	
*≥22*	--	*155 (53.26)*	
*BMI class for male* *[18.5 to 23)*	*125 (35.01)*	--	
*≥23*	*226 (63.31)*	--	
FEV_1_ predicted (%)	87.26 (83.58 to 90.02)	73.93 (69.01 to 77.61)	**<0.0001**
FEV_1_ predicted class *			
*<40%*	*26 (7.72)*	*35 (12.41)*	**0.049**
*[40% to 70%)*	*68 (20.18)*	*92 (32.62)*	**0.0004**
*[70% to 90%)*	*92 (27.30)*	*83 (29.43)*	0.54
*≥90%*	*151 (44.81)*	*72 (25.53)*	**<0.0001**
FVC predicted (%)	93.77 (90.54 to 95.69)	85.24 (81.63 to 88.72)	**<0.0001**
Sweat chloride (mmol/L) Pancreatic insufficiency *	78.44 (74.93 to 83.20) 133 (35.37)	76.00 (72.00 to 81.26) 91 (29.84)	0.27 0.13
Pseudomonas aeruginosa *	106 (28.42)	132 (43.00)	**<0.0001**
Staphylococcus aureus *	127 (34.89)	103 (34.45)	0.91

* data presented as *n* (%). In bold when statistical significance. In italic the data regarding the BMI and ppFEV_1_ classes.

**Table 4:** Characteristic of infertile males in 2018.

Characteristic	Male (*n* = 161) Median (Minimum to Maximum)
Age (years)	44.46 (24.46 to 64.29)
Age at diagnosis (years)	36.65 (19.93 to 56.08)
BMI (Kg/m^2^)	25.00 (17.48 to 36.08)
BMI class *	
*<18.5*	*1 (0.65)*
*[18.5 to 23)*	*41 (26.80)*
*≥23*	*111 (72.55)*
FEV_1_ predicted (%)	95.96 (28.72 to 147.03)
FEV_1_ predicted class *	
*<40%*	*2 (1.35)*
*[40% to 70%)*	*15 (10.14)*
*[70% to 90%)*	*36 (24.32)*
*≥90%*	*95 (64.19)*
FVC predicted (%)	97.67 (90.03 to 108.53)
Sweat chloride (mmol/L)	72.00 (9.00 to 150.00)
Pancreatic insufficiency *	36 (22.50)
Pseudomonas aeruginosa *	20 (12.58)
Staphylococcus aureus *	49 (31.41)

* data presented as *n* (%). In italic the data regarding the BMI and ppFEV_1_ classes.

**Table 5:** Difference between variables in according to sex (excluding infertile male patients) in 2018.

Characteristic	Male (*n* = 218) Median (95%CI)	Female (*n* = 311) Median (95%CI)	*p*
Age (years)	47.71 (45.38 to 49.87)	45.87 (44.38 to 48.13)	0.45
Age at diagnosis (years)	30.19 (28.12 to 32.64)	31.56 (30.48 to 32.97)	0.09
BMI (kg/m^2^)	23.51 (22.96 to 24.22)	22.22 (21.80 to 22.56)	**<0.0001**
BMI class for female and male *			
*<18.5*	*5 (2.45)*	*22 (7.56)*	***0.013***
*BMI class for female* *[18.5 to 22)*	*--*	*114 (39.18)*	
*≥22*	*--*	*155 (53.26)*	
*BMI class for male* *[18.5 to 23)*	*84 (41.18)*	*--*	
*≥23*	*115 (56.37)*	*--*	
FEV_1_ predicted (%)	76.83 (71.65 to 81.45)	73.93 (69.01 to 77.61)	0.31
FEV_1_ predicted class *			
*<40%*	*24 (12.44)*	*35 (12.41)*	*0.99*
*[40% to 70%)*	*53 (27.46)*	*92 (32.62)*	*0.23*
*[70% to 90%)*	*58 (30.05)*	*83 (29.43)*	*0.88*
*≥90%*	*58 (30.05)*	*72 (25.53)*	*0.28*
FVC predicted (%)	87.82 (84.04 to 93.14)	85.24 (81.63 to 88.72)	0.20
Sweat chloride (mmol/L) Pancreatic insufficiency *	86.80 (81.00 to 92) 97 (44.50)	76.00 (72.00 to 81.26) 91 (29.84)	**0.007** **0.0006**
Pseudomonas aeruginosa *	86 (39.81)	132 (43.00)	0.47
Staphylococcus aureus *	78 (37.14)	103 (34.45)	0.53

* data presented as *n* (%). In bold when statistical significance. In italic the data regarding the BMI and ppFEV_1_ classes.

**Table 6:** Genotype frequencies per CFTR2 categories in patients with borderline sweat test in 2018.

Genotype	*n* (%)
CF-causing/CF-causing	15 (13.27)
CF-causing/Variable Clinical Consequences	63 (55.75)
CF-causing/Unknown	21 (18.58)
CF-causing/NO CF	5 (4.42)
Variable Clinical Consequences/Variable Clinical Consequences	4 (3.54)
Variable Clinical Consequences/Unknown	3 (2.65)
Unknown/Unknown	2 (1.77)
Total	113 (100.00)
